# 2204: Evaluations of physiologic perturbations and their relationship with length of stay in neonatal hypoxic-ischemic encephalopathy

**DOI:** 10.1017/cts.2017.61

**Published:** 2018-05-10

**Authors:** Susan Slattery, Lei Liu, Haitao Chai, William Grobman, Jennie Duggan, Doug Downey, Karna Murthy

## Abstract

OBJECTIVES/SPECIFIC AIMS: Neonatal hypoxic-ischemic encephalopathy (HIE) is frequently accompanied with physiologic perturbations and organ dysfunction. Markers of these perturbations and their associations with length of stay (LOS) are uncertain. To estimate the association between changes in selected physiologic and/or laboratory values with LOS in newborns with HIE. METHODS/STUDY POPULATION: Using the Children’s Hospitals Neonatal Database (CHND), we identified neonates with HIE at our center born ≥36 weeks’ gestation from 2010 to 2016. Those with major congenital anomalies were omitted. Infants uniformly received therapeutic hypothermia for 72 hours unless death occurred sooner. Inpatient vital signs and selected laboratory markers were collected from our institution’s health informatics, electronic data warehouse (EDW) and then matched to records in CHND. With severity of HIE, gender, and confirmed seizures, each marker’s association with LOS was calculated using multivariable Cox proportional hazards regression equations. These analyses were stratified by mortality. Candidate markers were vital signs, pulse oximetry, creatinine, acidosis (pH), international normalized ratio (INR), and supplemental oxygen (FiO_2_). RESULTS/ANTICIPATED RESULTS: There were 66 eligible infants (38 males) and 1741 patient-days identified; Severe HIE (48%) and mortality (n=21, 32%) were common. Overall, the median length of stay (mLOS) was 20.5 days (25th–75th centile: 10–31 days), although shorter for nonsurvivors [nonsurvivors mLOS=8 days (5, 20); survivors mLOS=24 days (14, 31), *p*<0.001). Median birthweight and gestational age were 3.3 kg and 39.4 weeks’ gestation, respectively. In survivors (n=45, 1290 days), regression analyses demonstrated that none of the selected parameters were associated with LOS. Among nonsurvivors (n=21, 451 days), diastolic blood pressure changes [hazard ratio (HR)=0.93, 95% confidence interval (CI)=0.88, 0.97, *p*=0.04] was related to longer time of survival; conversely, temperature (HR=2.0, 95% CI=1.24, 3.26, *p*=0.005) was related to shorter survival. Creatinine, pH, INR, FiO_2_, or other vital signs were unrelated to time-to-death in nonsurvivors. DISCUSSION/SIGNIFICANCE OF IMPACT: In a pilot study of neonatal HIE, changes in physiologic values were related to duration of survival in nonsurvivors, while neither physiologic nor laboratory values were related to survivors’ mLOS. These results both exemplify novel uses for disease-specific, exposure-outcome relationships using EDWs and incorporates required functionalities of required software patches to extract, clean, and report from clinical information captured in electronic health records. We anticipate that text mining with techniques such as natural language processing will augment associations and/or predictions of short-term outcomes.

